# A case report of retroperitoneal pararenal Castleman’s disease associated with myasthenia gravis

**DOI:** 10.1186/1477-7819-12-331

**Published:** 2014-11-07

**Authors:** Shuai Wang, Shan-Wen Chen, Song-Liang Cai, Bai-Yan Jin

**Affiliations:** The fourth affiliated hospital of Zhejiang University School of Medcine, Shangcheng Road NO.N1, Yiwu, Zhejiang 322000 China; The first affiliated hospital of Zhejiang University School of Medcine, Qingchun Road NO.79, Hangzhou, Zhejiang 310003 China

**Keywords:** Castleman’s disease, Myasthenia gravis, Myasthenic crisis, Retroperitoneum, Surgery

## Abstract

**Background:**

Castleman’s disease (CD) is a relatively rare disorder characterized by the benign proliferation of lymphoid tissue. The combination of an occurrence of retroperitoneal pararenal CD with myasthenia gravis (MG) is extremely rare.

**Case presentation:**

The patient was admitted to our hospital for investigation of a retroperitoneal pararenal tumor which had been coincidentally diagnosed at a local hospital where he was admitted because of MG. The patient subsequently underwent an exploratory laparotomy and suffered from postoperative myasthenic crisis. Pathological examination revealed a left retroperitoneal mass of CD (hyaline vascular type). There was no recurrence of disease found after 7 months.

**Conclusions:**

CD with MG is a rare condition. Postoperative myasthenic crisis is a severe complication. The possibility of its occurrence must be in physicians’ minds and the risk of postoperative myasthenic crisis must be carefully considered when evaluating MG patients undergoing surgery.

## Background

Castleman’s disease (CD) was first described in 1956 by Benjamin Castleman, who identified a series of patients with solitary hyperplastic mediastinal lymph nodes containing small, hyalinized follicles and marked interfollicular vascular proliferation [1]. The disease is predominantly found in the mediastinum. Only 2% of CD cases have been located in the pararenal and retroperitoneal area [2]. The combination of an occurrence of retroperitoneal pararenal CD with myasthenia gravis (MG) is extremely rare. We report herein a rare case of CD in the pararenal retroperitoneum associated with MG of a 29-year-old man, who suffered from postoperative myasthenic crisis.

## Case presentation

**Figure 1:**
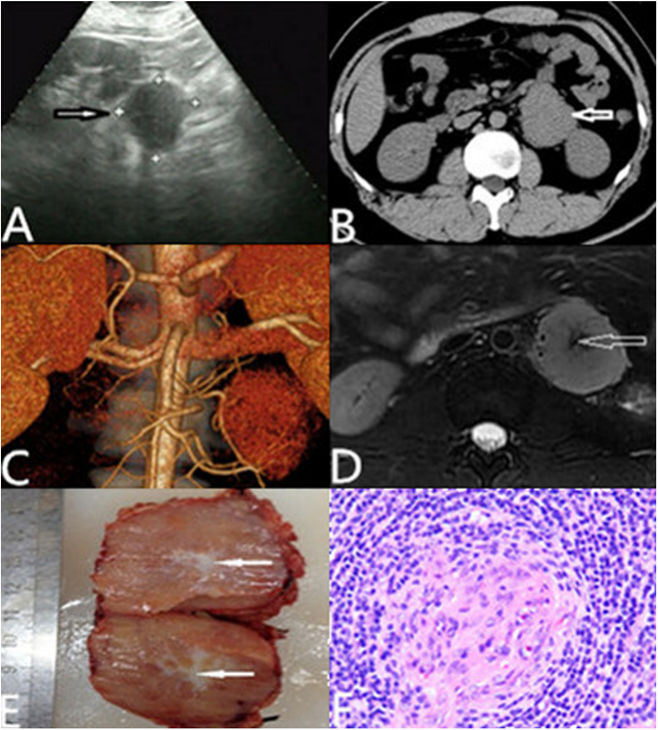
**Unicentric hyaline-vascular Castleman’s disease in a 29-year-old man. (A)** Ultrasonography demonstrated a low echogenic mass 5.4 × 5.2 cm in size (*arrow*) and there was no significant relationship with the left kidney. **(B)** Unenhanced CT of abdomen showed a well circumscribed, oval retroperitoneal pararenal mass (*arrow*) with homogeneous density. **(C)** Arteriograms showed arterial feeding to the mass from the abdominal aortic. **(D)** T2WI showed a slightly higher signal and the center of the mass displayed patchy low signal (*arrow*). **(E)** Photograph of a cut section of resected specimen demonstrated alternating reddish-brown and gray coloration. The reddish-brown areas represented cell components and the gray areas (*arrows*) represented fibrous tissue corresponding to the MRI findings. **(F)** Histological examination of the abdominal tumor (×400). Hyaline degeneration of small vessels with a wide surrounding by lymphoid follicle and lymphocytes arranged in an onion-skin pattern, confirming a diagnosis of Castleman’s disease. Immunophenotyping was positive for CD3, CD10, CD20, CD21, and CD23 antigens.

A 29-year-old man was admitted to our hospital on February 19^th^, 2014, for investigation of a retroperitoneal pararenal tumor. The tumor had been coincidentally diagnosed at a local hospital when he was admitted because of MG with a clinical manifestation of increasing upper and lower limb fatigue over 2 months. He was treated with acetylcholinesterase inhibitors (pyridostigmine at a dose of 3 × 30 mg) at the local hospital and clinical remission was achieved. A mediastinal magnetic resonance imaging (MRI) of local hospital did not show any evidence for thymoma. On admission, physical examination did not reveal any aberrations. Ultrasonography (US) demonstrated a low echogenic mass measuring about of 5.4 × 5.2 cm in size (Figure 1A). Unenhanced computed tomography (CT) scan of abdomen presented a well-encapsulated mass of homogeneous isodensity localized in the retroperitoneum (Figure 1B). CT arteriograms showed that the mass received its blood supply from abdominal aortic (Figure 1C). T1WI of MRI showed a homogeneous low signal, while T2WI and DWI demonstrated a slightly higher signal and the center of the mass displayed patchy low signal (Figure 1D). Electromyography exhibited that routine ulnar and median motor and sensory nerve conduction studies were normal, with no evidence for peripheral neuropathy; repetitive stimulation was positive, which supported a diagnosis of MG.

Preoperative biopsy for a definitive diagnosis was excluded in concern of the deep position and the possibility of hemorrhea. Preoperative embolization cannot achieve an efficient therapeutic result since the mass was not a hemangioma nor were there any vascular malformations. Although a definitive preoperative diagnosis was not possible, surgical excision was performed on February 25^th^, 2014. A mid-abdominal laparotomy revealed an oval mass with diffuse and rich vascularity located close to the lateral side of the abdominal aortic, near the anterior inferior part of the left renal. The surgical specimen was a well-circumscribed, encapsulated mass that measured 5 × 4 cm with a cut reddish-brown surface which changed to gray in the center (Figure 1E). An R0 resection was performed with a total blood loss of 200 mL without blood transfusion, and the operation time was 118 min. After 2 hours, the patient was sent back to the ward. However, 30 min later, he suddenly developed dyspnea with chest discomfort. Cyanosis appeared with clammy extremities after 2 min. The oxygen saturation dropped to 50 mmHg and cardiopulmonary resuscitation was implemented. Oxygen saturation then raised to 100 mmHg immediately, which proved that the dyspnea was caused by weakness of the respiratory muscles. The patient was transferred to the intensive care unit (ICU) after endotracheal intubation. A CT of the lung was performed on February 27^th^ showing no sign of pulmonary embolism except for pleural effusion. He was back to the ward on March 3^rd^ and discharged after 2 days. Histological examination of the abdominal tumor revealed angiofollicular lymph node hyperplasia of the hyaline-vascular type (Figure 1F). The patient came back to our clinic after one month and took a US which showed no recurrence of the disease. He received another US which also showed no abnormality 4 months after discharge. Further follow-up was made after 7 months and he lived free of disease.

## Discussion

The two clinical types (unicentric and multicentric) of CD have been sub-classified according to its histological forms: hyaline-vascular type, plasma cell type, and mixed type. Unicentric CD corresponds to the hyaline-vascular variant (>90%), and multicentric disease corresponds to the plasma cell variant. The unicentric type usually occurs in young people and is asymptomatic and associated with a benign clinical course, for which surgical excision is the most effective treatment. By contrast, multicentric CD is a systemic disease with lesions at two or more separate anatomic sites and is associated symptoms and laboratory abnormalities, such as anemia, fever, weight loss, night sweats, hypergammaglobulinemia, hypoalbuminemia, and elevated erythrocyte sedimentation rate, which need systemic therapy. CD cases associated with MG were mostly of the unicentric hyaline-vascular type, as was the case presented herein. A series of CD cases with MG have been previously reported [3–6], three of which were of the multicentric type. Ishikawa et al. reported that seven out of eight CD cases associated with MG were of the unicentric hyaline-vascular type, and the remaining one was of the multicentric mixed type [7].

CD is a great mimic and known to be associated with autoimmune diseases such as peripheral neuropathy, cytopenias, systemic lupus erythematosus, Sjogren’s syndrome, and MG. The overproduction of cytokines and interleukin is believed to play a central role in the development of CD and it is also associated with many clinical conditions related to immune disease [8]. The immune activity of CD may be the promoter of the antibody-mediated process of MG [4]. Klein et al. reported that the immune dysregulation of IL-6 has become the leading theory explaining the connection of CD and MG [9]. In CD, the overproduction of IL-6, a product of activated T-lymphocytes, results in increased B-cell differentiation with the emergence of plasma cell clones and subsequent formation of autoantibodies that initiate immunopathological reactions [10, 11]. Several studies have suggested that IL-6 is involved in MG, which would coincide with its activity as a promoter of B-cell differentiation and proliferation and an inducer of B-cell maturation into antibody plasma cells [12].

Less than 10 previous cases of CD associated with MG have been reported in the world literature with no evidence of hyperplasia or malignancy of the thymus in any of the cases. Only three of the cases have reported that the mass located in the retroperitoneum [3, 5, 13] and myasthenic crisis occurred in two [3, 13] out of the three, which means our case is the third one to date.

The main complication of MG is myasthenic crisis, characterised by a fast, marked decrease of muscle strength and compromise of bulbar muscles with dysphonia, dysphagia, and aspiration. The death rate following myasthenic crisis has drastically decreased after the 1950s from 80% to less than 10%, thanks to the use of early mechanical ventilation, admission to ICU, anticholinesterase drugs, steroid therapy, immunosuppression, and plasmapheresis [14–16]. Prompt treatment of myasthenic crisis leads to clinical improvement and can be life-saving. In this case, our rapid and effective rescue contributed to the uneventful survival and recovery of the patient.

## Conclusions

CD with MG is a rare condition. CD itself is a benign disease histologically, but postoperative myasthenic crisis is a severe complication. Therefore, the possibility of its occurrence must be remembered. The risk of postoperative myasthenic crisis must be carefully considered when evaluating MG patients undergoing surgery. Monitoring of post-operative vital signs can help in the early detection of complications.

## Consent

Written informed consent was obtained from the patient for the publication of this report and any accompanying images.

## Abbreviations

CD: Castleman’s disease
CT: Computed tomography
ICU: Intensive care unit
MG: Myasthenia gravis
MRI: Magnetic resonance imaging.
